# Supplementary material for: A deeper analysis in thyroid research: A meta-epidemiological study of the American Thyroid Association clinical guidelines
Source: PLoS One. 2020 Jun 10;15(6):e0234297. doi: 10.1371/journal.pone.0234297 (PMC7286515; doi:10.1371/journal.pone.0234297)
Supplement: S2 Table — (DOCX) [file pone.0234297.s002.docx]

| ***S2 Table.*** Paradigmatic Situations in Which Panels May Reasonably Offer Strong Based on Low Confidence in Effect Estimates | | | | | |
| --- | --- | --- | --- | --- | --- |
| **Situation** | **Benefits** | **Harms** | **Balance of Benefits and Harms** | **Examples** |  |
| 1. Life-threatening (or catastrophic) Situation. FOR | Low or very low | Immaterial (very low to high) | Intervention may reduce mortality in a life-threatening situation; adverse events not prohibitive. | A total lobectomy or total or near-total thyroidectomy should be performed in most patients with an incidental area of ATC within a DTC. This is based primarily on treatment recommendations related to the non-anaplastic component of the malignancy. |  |
| 2. Uncertain benefit, certain harm AGAINST | Low or very low | High or moderate | Possible but uncertain benefit; substantial established harm. | There are no credible scientiﬁc data to support the existence of ‘‘Wilson’s syndrome’’ and we recommend against the use of triiodothyronine escalation therapy for this indication, due to a lack of proven treatment beneﬁt and safety concerns relating to the risk of thyrotoxicosis. |  |
| 3. Potential equivalence, one option clearly less risky or costly. FOR | Low or very low | High or moderate | Magnitude of benefit apparently similar— though uncertain—for alternatives; we are confident less harm or cost for one of the competing alternatives. | There is no role for routine systemic adjuvant therapy in patients with DTC (beyond RAI and/or TSH suppressive therapy using LT4). |  |
| 4. High certainty in similar benefits, one option potentially more risky or costly. AGAINST | High or moderate | Low or very low | Established that magnitude of benefit is similar for alternative management strategies; best (though uncertain) estimate is that one alternative has appreciably greater harm. | Pregnancy is a relative contraindication to thyroidectomy and should only be used when medical management has been unsuccessful or ATDs cannot be used. |  |
| 5. Potential catastrophic harm. AGAINST | Immaterial (very low to high) | Low or very low | Potential important harm of the intervention, magnitude of benefit is variable. | In general, PTU should not be used in children. But if it is used, the medication should be stopped immediately and liver function and hepatocellular integrity assessed in children who experience anorexia, pruritus, rash, jaundice, light-colored stool or dark urine, joint pain, right upper quadrant pain or abdominal bloating, nausea, or malaise. |  |
| Total number of appropriate strong recommendations based on very low or low confidence in effects estimates. | | | | |  |
| 6. Best practice recommendation (for which sensible alternatives do not exist). | | | | Patients with TSH-secreting pituitary adenomas should undergo surgery performed by an experienced pituitary surgeon. |  |
| 7. Misclassification (The strong recommendation was warranted because the certainty of the evidence was actually moderate rather then low). | | | | None |  |
| 8. Lack of compelling of explanation (The recommendation should have been weak). | | | | Patients with symptomatic thyrotoxicosis due to painless thyroiditis should be treated with b-adrenergic-blocking drugs to control symptoms |  |
| Total number of strong recommendations based on very low or low confidence in effects estimates not consistent with paradigmatic situations. | | | | |  |
|  |  |  |  |  |  |

Table adopted from Bautista-Orduno KG, Dorsey-Trevino EG, Gonzalez-Gonzalez JG, et al. American Thyroid Association Guidelines are Inconsistent with GRADE - A Meta-Epidemiological Study. *Journal of clinical epidemiology.* 2020.
