# Supplementary material for: A deeper analysis in thyroid research: A meta-epidemiological study of the American Thyroid Association clinical guidelines
Source: PLoS One. 2020 Jun 10;15(6):e0234297. doi: 10.1371/journal.pone.0234297 (PMC7286515; doi:10.1371/journal.pone.0234297)
Supplement: Attachment — Submitted filename: Response to Reviewers.docx [file pone.0234297.s003.docx]

**Reviewer #1**

**Comment #1:**

This is an interesting and well written manuscript that highlights the reality of difficulties in reliance in evidence-based medicine and how much of what clinical guidelines (in almost every field of Medicine) recommend is based on less than optimal evidence and the need to constantly review and revise guidelines as new evidence emerges.
It would be very helpful (even if not the original intent of this paper) for the authors to highlight 3 specific areas or questions/recommendations in the field of thyroidology which would benefit from realistic RCT that would help advance the field. Many questions are not easily answered, others cannot be subjected to RCT for ethical reasons, etc

**Response:**

We appreciate the kind words and insightful observation of the reviewer. We recognize that the conduction of RCTs may be unfeasible due to funding, ethics, or other reasons, and that most of the times, the use of observational data is what is available as best evidence. Although it is impossible for this paper to provide a comprehensive review of current epidemiologic methods to draw causal statements from observational data, we have added a paragraph and several references that will guide the readers in case they are interested.

The manuscript reads as follows:

Based on our findings, we have found that most clinical recommendations, put forth by the ATA guidelines, are based on observational studies. This high prevalence may be due to several perks of thyroid research. For instance, conducting a multinational RCT requires for a multidisciplinary team that helps with the design, recruitment, analysis, and publication, and all these can only be afforded by proper funding. Yet, recent evidence has shown that thyroid research lacks the proper funding when compared to other areas with more prevalence and impact on morbidity and mortality.[31] This lack of funding impairs the proper conduction of RCT’s, and therefore, limits panelists to utilize the best available evidence to create clinical guidelines, despite the best stands for mostly being observational studies. But are observational studies always deemed to deploy low-quality of evidence? The answer is no.

There is an unquestionable valuableness of observational studies to yield causal results whenever conducted properly and adjusting for possible confounders.[32, 33] *Using observational data to make causal statements, however, requires the proper methods; causal questions requires causal method to answer them. For this, a rogue group of statisticians and epidemiologists defied the adage of “association is not causation” and stated that causality could be estimated from observational studies when proper methods are applied.[34-37] When this premises are violated, observational studies may yield an association between a treatment and an outcome even if this physiological plausibility fails to hold in real-world scenarios.* For instance, one of the most famous cases that bespeak the latter concept was the use of hormone replacement therapy (HRT) to prevent CVD in postmenopausal women.[38] The Framingham Cohort Study showed that the incidence of myocardial infarction (MI) among premenopausal women was virtually inexistent. This led to the assumption that by treating postmenopausal women with HRT, the incidence of MI will decrease.[38] Yet, this surmise failed when tested in a RCT.[39] Along this example, there are several other studies that highlight the precautions of using observational studies to provide care when analyzed incorrectly.[39, 40]

On the other hand, the conduction of an RCT’s are far from being exempt of bias.[41] Blindly inputting our faith towards everything that is labeled “randomized clinical trial” spurs for an inadequate application of evidence-based medicine to patient care.[42] A thorough and peruse appraisal of the evidence is necessary for clinicians to make confident clinical decisions and for panelists to develop trustworthy clinical recommendations. Additionally, some thyroid condition, especially rare diseases, with low prevalence, or high mortality, conducting a RCT would demand a tremendous effort, funding, recruitment, and some sort of fortunateness, and most likely, the resulting RCT will be underpowered, unrepresentative, and with misleading results. Observational studies may sometimes be the best and most feasible option to provide a causal inference and implement it to patient care.

**Reviewer #2**

**Comment #1**

It would be helpful to have a supplemental table/index of those recommendations that the authors found to be 'best practices' or for 'diagnostic' purposes. I was not clear from their methodology what they meant by that term and how it reflected in the guidelines chosen.

**Response:**

We have added a table as a supplementary file from a previous publication in which we provide examples from each case.

**Comment #2**

The authors discuss previous studies that have examined the pairing of strength of recommendation with quality of evidence; perhaps the authors could also elucidate which of the low-quality guidelines were associated with what strength of recommendation and if this correlated to the presence (or absence) of clinical trials.

**Response**

We appreciate the reviewer comment. However, classification based on the underlying quality was made solely on the recommendations, not the guidelines. Most guidelines had a mixed between low-, moderate-, and high-quality of evidence. We, however, acknowledge that it is important to elucidate what proportion of low-, moderate-, and high-quality recommendations had each guideline, and this has been elucidated in a previous publication from our same research group (Bautista-Orduno K. JCE 2020, doi: 10.1016/j.jclinepi.2020.02.010, PMID: **32145366**, https://www.jclinepi.com/article/S0895-4356(19)30836-4/pdf).

**Comment #3**

The authors encourages panelists on guidelines to fully endorse the GRADE guidance. I do not think this is appropriate in the context of this manuscript.

**Response**

We agree with the authors perception that the tone of our recommendations is strong. Hence, modifications to lower the tone were made.
